# Medical Gynæcology

**Published:** 1878-11

**Authors:** James H. Etheridge

**Affiliations:** Professor of Therapeutics and Forensic Medicine in Rush Medical College, Attending Gynæcologist to Central Free Dispensary; 603 Michigan Avenue


### Original Communications.

### MEDICAL GYNÆCOLOGY.

By J. H. Etheridge, A. M., M. D.

*Professor of Therapeutics and Forensic Medicine in Rush Medical College, Attending Gynæcologist to Central Free Dispensary.*

A common complaint made by young physicians, is, that they are unsuccessful in the local treatment of uterine diseases. This complaint is "common" because errors in, or omission of, proper treatment additional to local applications, is "common." Physicians who commence practice with the frequently-entertained, but exceedingly erroneous, idea, that the uterus is the *fons et origo mali* in all "female diseases," and that "local treatment" is *the* curative means to be instituted for curing them, will prove to be unsuccessful practitioners, or will very soon change their ideas of the pathology of these disorders, and change their treatment.

*It is exceedingly rare to find a so-called gynœcological case that does not present some serious departure from health in some organs or functions other than those peculiar to the sex of the patient.*

Any physician, unmindful of the universality of this fact, who will examine carefully in detail, in the first score of gynæcological cases, the *nervous, respiratory, circulatory, digestive and urinary systems*, and the general conditions presented by them of each patient, will be profoundly impressed with the idea that the symptoms of uterine disease are but a portion of the conditions demanding his attention. This is a point that, in gynæcology, has been much written upon in the past generation. No review of the opinions, on the relations of the disorders of the female generative organs to the general conditions referred to, is here intended. These opinions are accessible to any physician, who can read them himself. It may not be superfluous, however, to state that some authors have affirmed that the maladies of the female generative organs produce the general symptoms from which these patients suffer. Cure these generative maladies, and the general symptoms will be removed, they claim. Then, again, other authors have denied that gynæcological disorders produce general symptoms. These two classes of pathologists have written and argued over this point till severe things have been said of writers, till hard names have been called, till, in one instance, a professor went so far as to take another author's book into the lecture-room, and, before a class, to denounce the writer and his ideas, and then to tear up the book and, in a rage, to fling its fragments on the floor. As early as the beginning of this century gynæcologists had ranged themselves into two parties, which are distinct and separate to-day. They maintain their positions as resolutely as though they were dealing with a question of national finance or church polity, and nothing has budged them from their convictions.

As is usually the case in such contests, one party is right in some cases, and the other party is wrong; in other cases their positions are reversed, and, in still other cases, both parties are wrong, the truth lying midway between them. Every gynæcologist has seen cases where local treatment has cured the local trouble and removed the general symptoms. He has seen cases where protracted treatment has improved the uterine condition, and, not at all, or only very slightly, improved the patient as to general symptoms. Then, again, he has treated cases fruitlessly for a long time, when, upon resorting to medicines in addition to local applications, he has found his patient's condition improve at once. This good result, and this negative result from local treatment used alone, I have seen so often and so discouragingly, that, for investigation, I have been in the habit for a long time, through the efficient assistance of Dr. B. W. Griffin, of taking very complete histories of my cases at the Central Free Dispensary. What this work may lead to I am unable to predict, but I can say that it has modified my practice to such an extent that the administration of medicines forms a large part of the treatment used. Where an alimentary canal trouble, as indigestion, costiveness, constipation, etc., exists, medicines are administered to correct this departure from health. Where rheumatism or syphilis or scrofula, anæmia, or that Protean monster, the gouty vice, is met with, appropriate remedies are given. It matters not whether a disorder be met with in the *nervous alimentary*, *respiratory*, *circulatory*, *renal* or *cutaneous system*, some remedial measures, in all gynæcological cases, besides local treatment, must be resorted to.

To illustrate the universality of general symptoms in gynæcological patients, 50 cases, as they came to the dispensary, are herewith presented. They are not selected—they are consecutive cases. Fifty only are presented, because that number illustrates the end sought as well as fifty thousand cases would illustrate it. These patients are such as are met with in a large cosmopolitan dispensary and are, of course, from among our poorer classes, acquainted with hard work, privations, scant tables and garments, and, in many instances, with an unbroken series of years of either gestation or suckling. These cases, taken from the case sheets numbered consecutively, ran back from date—August 20, 1878, till the requisite number has been reached.

Case taking has been followed, in this dispensary work, with rigid uniformity. First, is annotated what the patient of her own accord complains of; in most instances her own words are used, enclosed in quotation marks. It is instructive to notice the universality of abnormal sensations described. A pain somewhere is invariably complained of. After the patient has delineated her complaints, the real work of case taking is begun. The various systems of the human organism are carefully questioned in topographical order, from above downwards. First the *nervous* system is examined, second the *alimentary*, third the *respiratory*, fourth the *circulatory*, fifth the *renal*, sixth and lastly, the *cutaneous*. Under the nervous symptoms are noticed all pains and abnormal sensations arising from any cause whatever. The more elaborate means used in questioning the nervous system were not resorted to.

Under all of the other divisions is annotated whatever is found that can be properly classed thereunder. Occasionally a symptom is found which can be classed under one of two divisions—*e. g.*, *vertigo*, which may be be considered a *nervous* or a *circulatory* symptom.

Following this, is an examination of heredity, family diseases and idiosyncrasy. Next, the patient is examined with regard to the functions and organs peculiar to her sex, menstruation, abnormal vaginal discharges, and the condition of the uterus and its annexæ. It will be observed that the last class of symptoms is entirely omitted from the table herewith presented. Only those symptoms are given which are observed aside from the gynæcological aspect of the cases:

| No. of Case and Diagnosis. | Nervous. | Alimentary. | Respiratory. | Circulatory. | Renal. | Cutaneous. | Remarks. |
|---|---|---|---|---|---|---|---|
| CASE 1. Endocervicitis. | Frontal and occipital cephalalgia. Lumbar rachialgia. Thoracic muscular pains. Pains in epigastrium, hypogastrium and ovarian regions. | Tongue coated white. Bad taste in mouth mornings. Gastric indigestion. Tympanites. Constipat'n (tri-weekly evacuations). Occasional hæmorrhoids. | Chronic post-nasal catarrh. | | Urine deposits reddish brown sediment. | | |
| CASE 2. Endocervicitis. | Frontal, coronal and occipital cephalalgia. General arthritic pains Burnings in soles of feet. Numbness of hands and forearms. | Bad taste in mouth mornings. | Chronic post-nasal catarrh. | Frequent palpitation. Vertigo occasionally. | Urine deposits heavy mucous precipitate. Urinates far too frequently. | Has brown spots under skin of face. | Says she "has had rheumatism for years." |
| CASE 3. Subinvolution. | | Foul breath and very bitter taste in mouth mornings. Anorexia mornings. Tympanites evenings. | | | | | Supposed cause is her last labor. |
| CASE 4. Split Cervix (double) Hyperplasia. | Frontal and occipital cephalalgia. | Thickly coated tongue, white. Bad taste in mouth. Gastric indigestion. Tympanites. Left lobe of liver enlarged. | | | Urination followed by pain along ureters for two or three minutes. | | |
| CASE 5. Hyperplasia and catarrh of uterus. | "Rheumatism" in the shoulder. Lumbar and sacral rachialgia. | Brown tongue. Bitter taste in mouth. Anorexia. Obstinate constipat'n always demanding cathartics. | | Occasional vertigo. | | | Stands erect with difficulty. Walking or riding produces great pain in lumbar region. |
| CASE 6. Uterine Catarrh and Hyperplasia, and urethral vascular tumor. | Coronal and occipital cephalalgia. Sacral rachialgia. Frequently has shooting pains all over. | Gastric indigestion. Tympanites. Constipation. | | Occasional palpitation and vertigo. | "Passes water fifty times a day" and three or four times every night. Urine deposits uric acid. | | |

| No. of Case and Diagnosis. | Nervous. | Alimentary. | Respiratory. | Circulatory. | Renal. | Cutaneous. | Remarks. |
|---|---|---|---|---|---|---|---|
| CASE 7. Endometritis and endocervicitis. | Frontal Cephalalgia. Lumbar rachialgia. | Tympanites. | | | | | |
| CASE 8. Endocervicitis. | Occipital, coronal and frontal cephalalgia. Thoracic, lumbar and sacral rachialgia. | Bad taste in mouth mornings. Occasional vomiting. Gastric dyspepsia. | | Exertion produces palpitation. | | | Often has muscular rheumatism with hyperæsthesia. |
| CASE 9. Spammenorrhœa | Temporal cephalalgia on both sides. | Bad taste in mouth mornings. Yellow tongue. Anorexia by spells. Constant tympanites. Cannot eat eggs. Craves acids. | Exertion produces dyspnœa. | Exertion produces palpitation. Cold feet. | | | Has had rheumatism. |
| CASE 10. Amenorrhœa. | Constant frontal and temporal headache. | Bitter taste in mouth mornings. Obstinate constipation. | Exertion produces shortness of breath. | Palpitation produced by fright or exertion. | Lithic acid deposit in urine. Occasional pain over kidneys. | | Has never menstruated. Has menstrual prodromata about middle of every month. Aged 17 years. |
| CASE 11. Endocervicitis. | Coronal cephalalgia. Lumbar rachialgia. | | | | | | Had an indurated chancre in June, 1877. Says that every three or four weeks has sore throat unconnect'd apparently with menstruation, which occurs every four weeks. |
| CASE 12. Endometritis and endocervicitis. | Frontal cephalalgia. Sacral rachialgia | Bad taste in mouth mornings. Tympanites and constipation. | | Occasional palpitation. Face flushes often and mind at same time becomes confused. | Lithic acid and mucous deposits in urine. Often feels too weak to hold her water. | | Is sucklinga babe. |

| No. of Case and Diagnosis. | Nervous. | Alimentary. | Respiratory. | Circulatory. | Renal. | Cutaneous. | Remarks. |
|---|---|---|---|---|---|---|---|
| CASE 13. Split cervix and general hyperplasia of uterus. | Coronal cephalalgia. Weight at back of head. Lumbar rachialgia. | Gastric and intestinal indigestion. Great pain precedes bowel evacuations. | | Occasional palpitations. Has cold feet a great deal. | Urine dep sits mucus and lithic acid. Some days passes it every hour. | | Locomotion exhausts her greatly. Can scarcely stand erect upon arising mornings. |
| CASE 14. Endocervicitis and chronic vaginitis. | Frontal and coronal cephalalgia. Has a sick headache every 4 weeks. Has hot and cold flashes all over. | Teeth reduced by caries to roots and stubs. Gastric and intestinal indigestion. Constipation. | Coughed a great deal, first and last. | Palpitation every day. Cold feet and legs. | | | |
| CASE 15. Ante-flexion. chronic endometritis and endocervicitis. | Muscular pains and occasional stiff joints. | | Coughs and raises a little mornings. Bronchial congestion. | | Urine deposits lithic acid. | | |
| CASE 16. Endocervicitis. | Lumbar rachialgia. Vertigo. | Bad taste in mouth mornings. Obstinate constipation. | | Cold extremities. | | | |
| CASE 17. Metrorrhagia. | Coronal cephalalgia. Thoracic rachialgia. | Bad taste in mouth mornings. Gastric and intestinal indigestion. Constipation. Has "summer complaint" every 2 or 3 weeks. | | Much palpitat'n and cold extremities. | | | Locomotion produces palpitation, trembling and pains in legs. |
| CASE 18. Subinvoluti n. | Occasional frontal cephalalgia. Sacral rachialgia, exaggerated during menstruation. | Tympanites prior to menstruation. | | | | | Is spammennorrhœic. Pains in right knee 3 or 4 days before menstruation. |
| CASE 19. Endocervicitis. | Coronal cephalalgia. | Bad taste in mouth mornings. Gastric dyspepsia. | | | | | Suckling a babe. Is constantly tired. |

| No. of Case and Diagnosis. | Nervous. | Alimentary. | Respiratory. | Circulatory. | Renal. | Cutaneous. | Remarks. |
|---|---|---|---|---|---|---|---|
| CASE 20. Metrorrhagia. | Coronal cephalalgia. | Very yellow tongue. Anorexia. Occasional tympanitis. | | Occasional palpitation and vertigo. Cold extremities. | | | Has had two attacks of rheumatic fever lasting 9 and 5 months respectively. |
| CASE 21. Prolapsus of bladder. | Coronal and occipital cephalalgia. Lumbar rachialgia. | Capricious appetite. Occasional gastric indigestion. Constant tympanites. Constipation. | | Freqnent palpitation of heart. | | | Is of the rheumatic diathesis. |
| CASE 22. Chronic Endometritis and chronic vaginitis. | Frontal, coronal and occipital cephalalgia. Constant lumbar rachialgia. | Bad taste in mouth mornings. Gastric dyspepsia. Tympanites and constipation—once went 24 days. | | | Urine leaves a white precipitate upon cooling. | | Has had repeated ischio-rectal abcesses. Has now 3 or 4 fistulæ. |
| CASE 23. Endometritis. | Coronal cephalalgia. Occasional migraine. Pain encircling body through ovaries and sacrum. | Foul tasting mouth mornings. Gastric dyspepsia. | | | | | Passed menopausis 1 year ago. |
| CASE 24. Amenorrhœa. | | | | Vertigo for 3 days, a great deal at menstrual epoch. | | | Is 16 years old. Has menstruated only 3 times, Dec., Jan. and Feb., last time being 3 months ago. Took cold and has not menstruated since. |
| CASE 25. Endocervicitis. | Temporal cephalalgia. Pain across epigastrium. Lumbar rachialgia. | Always bad taste in mouth mornings. Cheese always produces acute dyspepsia. | | Suffocation spell always follows the extinguishing of light at night, without which she cannot sleep. | | | |

| No. of Case and Diagnosis. | Nervous. | Alimentary. | Respiratory. | Circulatory. | Renal. | Cutaneous. | Remarks. |
|---|---|---|---|---|---|---|---|
| CASE 26. Subinvolution and Uterine Catarrh. | Much of frontal, and an occasional coronal cephalalgia and lumbar rachialgia. Frequently has "fearfully nervous spells." | Foul taste in mouth mornings. Gastric indigestion. Tympanites since childhood. Always constipated. | | Pulse intermittent. Has cold feet. | Lithic acid and mucous deposit in urine. | | Has had 6 abortions within the past thirty months. |
| CASE 27. Amenorrhœa. | | Appetite poor. Gastric dyspepsia. Costiveness. | | Exaggerated cardiac action. No organic heart disease. | | | Aged 15 years. First 4 menstruations were regularly two months apart. Since then she is unwell every three months regularly. |
| CASE 28. Amenorrhœa. | Coronal cephalalgia. | Gastric indigestion. | | | | | |
| CASE 29. Mucous Polypus (small) and endocervicitis. | Frontal cephalalgia. Hot, boring, sacral rachialgia. | Bad breath. Bad taste in mouth mornings. Flatulent dyspepsia. Great tympanites before menstruation. | | | | | |
| CASE 30. Retroflexion and endometritis. | Occasional headaches. Lumbo-sacral rachialgia. Has "frequent nervous spells." | | | | Profuse polyuria after nervous spells. | | |
| CASE 31. Endometritis and "ulceration." | Neuralgic headache in temples and eyes frequently. Pain down left sciatic nerve during menstruation. | Bad taste in mouth mornings. Extreme constipation, sometimes 14 or 15 days. | | | | | |
| CASE 32. Endometritis. | Great pain in temples at menstrual epoch. Sacral rachialgia. | Bad taste in mouth mornings. Obstinate constipation. | | | | | |

| No. of Case and Diagnosis. | Nervous. | Alimentary. | Respiratory. | Circulatory. | Renal. | Cutaneous. | Remarks. |
|---|---|---|---|---|---|---|---|
| CASE 33. Endometritis. | Continuous cephalalgia, frontal or occipital. Sacral and lumbar rachialgia. | Yellow tongue. Carious teeth. Bad taste in the mouth mornings. Anorexia. | | | | | Locomotion produces excessive fatigue. |
| CASE 34. Endocervicitis. | Temporal cephalalgia before menstruation or upon becoming fatigued. Sacral rachialgia | Poor appetite Constipated habitually. | | | | | |
| CASE 35. Endometritis. | Coronal cephalalgia. Sacral and lumbar rachialgia. | Carious teeth. Yellow tongue. Bad taste in mouth mornings Gastric dyspepsia. Constipation. | Chronic bronchitis, with profuse expectorations. | Palpitation and dizziness upon exertion. | Lithic acid deposited in urine. | | |
| CASE 36. Endocervicitis. | Coronal cephalalgia. Sacral rachialgia. Pain in both groins. | Anorexia. Gastric dyspepsia. Constipation. | Post-nasal catarrh. | Momentary dizziness coming on frequently. | Lithic acid deposit. | | |
| CASE 37. Endometritis. | Coronal cephalalgia. Sacral rachialgia. Pain in groins upon exertion. Submammary pains. | Poor appetite. Constipation. Occasional hemorrhoids. | Out of breath easily upon exertion. | Palpitation upon exertion. | Uric acid deposited in urine. | | |
| CASE 38. Subinvolution. | Frontal and coronal cephalalgia. Sacral rachialgia. Pains in hypogastrium, and up and down the thighs. | Anorexia. Gastric indigestion. Constipation | | | | | |
| CASE 39. Subinvolution. | Frontal cephalalgia. Much pain in hypogastrium and over sacr'm. | Gastric and intestinal indigestion. | | | Has had attack of gravel for 2½ years. Passes bloody urine after the attacks. | | |
| CASE 40. Endocervicitis. | Much pain all along the spine, excepting when pregnant. | | | | | | Menstruates very irregularly. Used to h've terrible dysmen'rhœa prior to 1st pregnancy. |

| No. of Case and Diagnosis. | Nervous. | Alimentary. | Respiratory. | Circulatory. | Renal. | Cutaneous. | Remarks. |
|---|---|---|---|---|---|---|---|
| CASE 41. Endocervicitis. | Daily temporal and occipital cephalalgia. Rachialgia, thoracic, lumbar and sacral. | Appetite variable. Gastric dyspepsia. Constipation. | | | Urine deposits lithic acid. | | |
| CASE 42. Rectocele. | Severe coronal cephalalgia. Pains in left side and groin, and left thigh to knee. | Anorexia. Gastric dyspepsia. Obstinate constipation. | Easily out of breath. | Palpitation upon exertion. | Lithic acid deposit in urine. | | |
| CASE 43. Subinvolution. | Extreme coronal cephalalgia. Sacral rachialgia. Pains in hypogastrium. | Appetite very capricious. Gastric dysdepsia. Constipation. | | Palpitation daily. | Uric acid deposit in urine. | | Menstruated first at 20 years of age. |
| CASE 44. Cervicitis and endocervicitis. | Daily very severe coronal and temporal cephalalgia. Pain along the whole of the spine. | Appetite very ch'geable. | Easily put out of breath. Post-nasal catarrh. | | Urine deposits lithic acid in great abundance. | | |
| CASE 45. Hyperplasia with endometritis. | Great lumbar rachialgia. | Acid and flatul'nt dyspepsia. Vomiting very common and distressing. Almost starved on account of dyspepsia. | | Palpitation. | | | |
| CASE 46. Subinvolution. | Burning coronal cephalalgia. Pain in lumbar and sacral regions. | Anorexia in morning. Nausea. Gastric dyspepsia. Costiveness. | | | Urination produces smarting and scalding sensations. | | |
| CASE 47. Hypertrophy of cervix and endocervicitis. | Coronal and occipital cephalalgia. "Sick headache." | Anorexia. Constipation. | Easily put ont of breath. | Exertion causes palpitation. | | | |
| CASE 48. Subinvolution. | Pain in frontal and coronal regions a great deal. Much pain in hypogastric and inguinal regions. | Anorexia. Gastric dyspepsia. Defecation always produces great pain. | | Vertigo frequently. | | | |

| No. of Case and Diagnosis. | Nervous. | Alimentary. | Respiratory. | Circulatory. | Renal. | Cutaneous. | Remarks. |
|---|---|---|---|---|---|---|---|
| CASE 49. Subinvolution. Retroversion and metrorrhagia. | Pains in both groins and lumbar region. | Intestinal dyspepsia. | | | Urinates too often. | | |
| CASE 50. Subinvolution. | Hypogastric pains for two years past. | Great tympanites. | | | | | Very spammenorrhic. Is very easily used up by exertion. |
| CASE 51. Cervicitis and left lateroflexion. | Lumbar rachialgia. | Has gastric indigestion. | | Palpitation very much. | Uric acid deposit in urine | | Is continually languid and low spirited. |
| CASE 52. Subinvolution, merorrhagia and utertne catarrh. | Frontal cephalalgia. Lumbar rachialgia. Groin pain during menstruating. | Gastric dyspepsia. Tympanites. | | | | | |

The first thing especially noticeable in the foregoing table is the uniformity of nervous and alimentary symptoms. The more important of the two are the alimentary derangements, because they invariably produce abnormal sensations, in some instances leading neurologists to make some astonishingly learned but frequently erroneous diagnoses. The stickler for exactitude of knowledge of the pathological relations in these cases, will at once begin to query as to whether the uterine disorder cause the alimentary disorder, or whether cause and effect are the reverse of this, and thus at once plunge into the midst of the old, old discussion from which, as yet, but very little of lasting good has been evolved. The writer wishes to call urgent attention to the necessity for special medical treatment of all cases of alimentary derangements, met with in gynæcological cases, irrespective of what produces them, accepting the fact of their existence as an indication for special treatment. This, of course, makes every gynæcologist a careful inspector of every factor and function of alimentation. He must, of necessity, examine the teeth, tongue, stomach, liver, spleen, intestines, rectum, ascertain if the food be well chewed, be properly cared for in the stomach, and supplied with the necessary digestive juices in the intestines, and if the *refuse material* is freely and regularly evacuated from the system. If 1,000 consecutive, miscellaneous dispensary patients could be examined with care and exactitude, and the universality of alimentary derangements pointed out, much surprise would be excited that there are not specialists devoted to the consideration of alimentary maladies. Defective digestion produces poor blood, and one of the accompaniments of an inferior quality of blood is a variety of abnormal nervous sensations or manifestations, varying according to the temperaments or idiosyncracies of the different patients. If this defective digestion and impoverished blood in gynæcological cases exist for months or years, cases whose long duration we see daily, we are sure to see evidences of some profound neurologic lesion capable of being christened by some ponderous name. Then these patients are said to have some nervous disease and are pronounced permanent invalids. One will be surprised frequently to see how quietly nervous symptoms will gradually disappear upon the correction of a dyspepsia, or the removal of a costiveness or a constipation. Every physician will acknowledge that a patient constantly carrying around with her a load of excretion, indicated by the muddy sclerotic, foul tongue, bad taste in the mouth mornings, icteric, lifeless skin, sluggish bowels and loaded urine, can no more produce good blood and hope to be free from multifarious pains, than she can hope to pass through confinement painlessly. Yet the majority of gynæcological cases present this condition with an astonishing uniformity. The longer they have had uterine disease, the more deeply is this condition intensified. Every physician also knows that he seldom sees a case of uterine disease demanding treatment, where the patient presents a bright eye, a clear tongue, a faultless digestion, no pains, elastic step and a vivacious manner. Could every case be followed from the day that the patient first departed from the line of the exact physiological function of all of the organs of her system, it would readily be comprehended how and why it is that it is almost impossible to meet with a woman perfectly healthy. Over forty years ago Marshall Hall called the attention of the profession to the prime necessity for promoting excretion from the system, thus preventing auto-inoculation with excretion poison, a source of a large proportion of ailments which physicians treat.

We all know the peculiarly vicious, sickening odor of the breath of an habitually costive patient, whose absorbents are incessantly sucking up from the colon its fæcal load and carrying it into the circulatory torrent, whence it seeks other outlets, notably the lung and skin, producing bad breath and a lifeless looking skin. Such patients are pergrinating privies, endeavoring constantly to defecate through the lungs and skin. Such excessively bad blood as these patients make, will supply their nerve centers with just the right sort of pabulum to ensure abnormality of function, and we will be called upon to prescribe for various pains, for a functionally disordered circulatory system, and for the various diseased conditions of the generative organs. I am convinced that some of the painful conditions of the ovaries so commonly met with, are produced by the impoverished blood that such patients present. I recall especially one dysmenorrhœic patient from among the many that I have treated, who had suffered almost infinitely from "inflammatory" disorder of the right ovary. She had been blistered, cupped, unguented variously over the ovary; had had local treatment, and had had the most approved and atrocious varieties of treatment during several years, from various physicians, and continued to successfully and satisfactorily grow worse. When called to her, I found her bed-ridden, a condition that she had then enjoyed for five months. The most excruciating tenderness of the ovary was elicited by pressure. Assuming the upright position, and especially walking, greatly increased her pains. Menstruation was an event which totally capped the climax of her sufferings, which were then so terrible as to necessitate intoxicating amounts of gin or anæsthesia from chloral or chloroform. When summoned to see the patient, I was informed that I was called to "prescribe for a probably forming pelvic abcess," another physician having so pronounced it. I found her as above described, with many other symptoms. She had a constant cephalalgia, a most rebellious constipation, excreted a very large amount of uric acid, and was anæmic to a moderate extent. The constipation and anæmia were soon done away with, but a large amount of uric acid was still noticeable. Recognizing the lithæmic vice, the patient was put upon colchicum and citrate of potassium. Slowly she improved, the ovarian tenderness disappeared, and the dysmenorrhœa became comfortable enough to endure by simply remaining in bed. I first saw and prescribed for this patient three years ago, and in six months she was able to go without intoxicants or analgesics during menstruation, to walk and ride and to spend the day out of bed. Since that date she has taken the various anti-lithic remedies *pro re nata*. and has maintained a satisfactory condition of health, having made journeys and resumed her former occupations. From first to last I used no local treatment whatever. The removal of excretive matter from the system and the improving digestion to the extent of allowing uric acid to be oxidized to the dignity of urea, and thus annihilating the lithemia, which seemed to completely saturate her system, gave her system a pure blood and removed her sufferings. In this case medical gynæcology seemed to wholly answer my purpose.

Thus far I have spoken only of remedies for alimentary disorders. With regard to the respiratory and circulatory functional disorders, it may be added that, to a large extent, they are dependent upon imperfect alimentation. The same may be said of so-called renal disorders. The lithic acid diathesis is commonly associated in the medical mind with kidney derangement, whereas it is *wholly* dependent upon defective alimentation. Perfect digestion will always secure the proper proportions of urinary constituents. Colchicum and potash, vaunted so highly in lithiasis, the former for exciting the kidneys to "remove uric acid from the system," and the latter for "dissolving uric acid out of the blood," are now thought to exert their good effects *wholly* upon the ferments of the alimentary canal juices, and thus to promote perfect digestion.

When we reflect that so large a proportion of the gravest disorders of the human system, as anæmia, lithæmia, rheumatism, rickets, scorbutus, scrofula and tuberculosis can be more or less directly traced to the imperfect construction of good blood; when we recall that the latest conviction in the minds of epidemiologists concerning the liability of human beings to be stricken down by epidemics is, that they go soonest who are in the lowest grades of general health, although engaged in their usual avocations, and, that they who are in a perfectly sound physiological condition seldom or never fall before epidemics; when we consider that the so-called "predisposing cause" of disease resident in everybody can be, when eliminated from erroneous theories, brought down to the then existing condition of the blood, and made conspicuous and powerful in proportion to the deteriorated condition of this vital fluid, we can see the constant necessity that physicians are under to see to it that their patients should make the best of blood. Inspection of the latest works on practice all point to the one idea underlying all treatment, that good blood is a *sine qua non* to a return to health.

Two or three objections may be raised by physicians to the method of practicing gynæcology so evidently indicated herein. One objection is, that prescribing for the various derangements found in cases of female maladies is nothing more nor less than *general prescribing.* The admission is candidly made that this claim is a solid and commendable one. If the outlook of gynæ-

cology as a science is to be obscured by the danger of prescribing for all of the symptoms that its cases present, then is it so much the worse for this "science." It must *as* candidly be admitted that, in so far as gynæcologists can separate this so-called "science" from the general science of medicine and surgery, will they be able to obfuscate the minds of the ordinary medical practitioner with the error or heresy that gynæcological cases need *special* care and skill, and that they thus pass beyond the necessity for general prescribing. That physician is the safest gynæcologist who fails not to lose sight of the fact that the good health of women is but the other name for the perfect performance of function in her *nervous*, *alimentary*, *respiratory*, *circulatory*, *renal and cutaneous* systems; and at the same time that he judiciously makes local applications, he *as* judiciously prescribes appropriate remedies for whatever symptoms are presented for his consideration.

Of course the consideration of the treatment of these symptoms or conditions cannot be here taken up, for it would necessitate the writing of a small volume on practice. The only guide to follow is a carefully digested knowledge of physiology, pathology and therepeutics. These teach us the inseparableness and inter-dependence of the organic functions, the unification and harmonizing of which is the *only* thing that a physician ever strives to attain in any patient.

Another objection that can be offered is, that using local treatment and, at the same time, resorting to general prescribing, tend to ignore the differentiating between local and general diseases. This objection, repeatedly advanced, savors so offensively of prejudice and ignorance, in that it compels one to announce that he arrays himself upon the side of one or the other of the two factions in the ranks of gynæcologists spoken of early in this paper, that it reminds one of Sangrado's fixedness of theory of bleeding in the measles. The men raising this objection seem *determined* to have a theory to practice in accordance with. They seem, apparently, to consider themselves incapable of practicing unless they can tell precisely which first departed from the healthy standard, the generative organs or the general system. So long as they *will* have a theory, and *will* practice in accordance therewith, and *will* fail to cure some easily curable cases because of their dogged determination to play Procrustes in medicine, why they must be allowed to frame theories, to stick to them and to make brilliant failures.

After all that has been said thus far, the question may arise what are the results of administering medicine along with local treatment to gynæcological patients? Dispensary practice is, perhaps, not the best field in which to observe results. Patients come once and physicians are never sure of their return. However those who have returned for continued treatment, present a percentage of improvement overshadowing that obtained before general medication was used, to such an extent that I have been astonished.

In addition to correcting alimentary disorders (a class of troubles present in at least nine out of ten patients treated), and other functional derangements wherever found, I have made very free use of ergot fluid extract. In all cases of recent uterine enlargement from whatever cause, or of endometritis, or in any case presenting profuse catarrhal discharge, ergot does good. One of the first remarked good results of it, is the diminution of the various pains about the pelvic region. I frequently give ergot alone for a week before examination or treatment, and it is common to hear of the above mentioned good results. (I hope to be able to present, at no distant day, some points upon the initial use of ergot in gynæcology that shall be of great service by way of aiding in diagnosis.) Enlargement from hyperplasia is but little affected *permanently* by ergot. Enlargement from recent labor or abortion, *especially if accompanied by* PROFUSE LEUCORRHŒA, is in nearly all cases relieved *without local treatment*, by free catharsis, quietude and ergot used largely. Nearly all cases of inflammatory uterine disorders having metrorrhagia as a common accompaniment, will be astonishingly benefited by ergot used continuously, till cured. I particularize thus concerning ergot because it is so largely useful in these maladies. It cannot be taken by all patients. Some women are so dreadfully nauseated by it that its use has to be abandoned. A few patients can bear ergotine who cannot bear ergot fluid extract. Still there are others who cannot take ergotine.

In urging the claims of Medical Gynæcology, the writer wishes to be distinctly understood as desiring to advocate a recurrence to the good old-fashioned way of regarding *all* diseases as manifestations of the disturbance of relations between *waste* and *repair*, an equality of which means HEALTH. This idea is older than any physician living to-day. In the modernized craze for refinements in etiology, pathology, diagnosis, and for mutiplicity of specialties, this simple and trite fact is too often overlooked. If enquiry of each system of the human organism reveal any departure from health, let the physician busy himself with correcting it. He should never lose sight of the utter, the fantastic absurdity of supposing that there can be an overshadowing necessity for avoiding medication for the almost invariably accompanying functional disorders presented in uterine maladies, as is only too commonly done by physicians to-day.

603 MICHIGAN AVENUE.

